# Induction of Apoptosis by Matrine Derivative ZS17 in Human Hepatocellular Carcinoma BEL-7402 and HepG2 Cells through ROS-JNK-P53 Signalling Pathway Activation

**DOI:** 10.3390/ijms232415991

**Published:** 2022-12-15

**Authors:** Xiang Wang, Sen Zhang, Keyan Han, Lisheng Wang, Xu Liu

**Affiliations:** Medical College, Guangxi University, Nanning 530004, China

**Keywords:** liver cancer, ZS17, ROS, JNK, mitochondrial dysfunction, apoptosis

## Abstract

Hepatocellular carcinoma (HCC) is one of the most common malignancies and ranks third among cancer-related deaths worldwide. Using matrine as a lead compound, 12 matrine derivatives were designed and synthesised, and their antiproliferative activities were evaluated in four cancer cell lines. Eight of the twelve compounds showed strong antiproliferative activity, with an IC_50_ of <10 μM. The compound **ZS17** exhibited strong antiproliferative activity in hepatocellular carcinoma cell lines with IC_50_ values in the range of 3.014–3.388 μM, which was much lower than that of matrine. Furthermore, we explored the role of **ZS17** in inducing apoptosis in HCC cells in vitro and in vivo, as well as possible mechanisms involved. **ZS17** inhibited the proliferation of BEL-7402 and HepG2 cells in time- and dose-dependent manners. In addition, we found that **ZS17** significantly induced apoptosis and ROS (reactive oxygen species) production, promoted JNK phosphorylation, activated p53, and activated the caspase signalling pathway. Furthermore, the antioxidant NAC, JNK inhibitor SP600125, and Si-JNK increased cell viability, re-established cell metastasis, and inhibited **ZS17**-induced apoptosis. An in vivo antitumour assay demonstrated that **ZS17** significantly reduced the number of migrating HepG2 cells in zebrafish embryos and suppressed the growth of HepG2 xenografts in nude mice without any obvious side effects. Our study demonstrated that the ROS-JNK-P53 pathway plays an important role in the destruction of liver tumour cells by **ZS17**. Thus, **ZS17** may represent a promising chemotherapeutic agent for the treatment of HCC patients.

## 1. Introduction

Liver cancer is the most common cause of cancer-related deaths worldwide and the third-most common cancer in the United States [1]. In the United Kingdom, only 20% of patients with the disease remain alive one year after diagnosis. There is a higher prevalence of liver disease in developing countries [2]. Clinically, patients usually undergo surgical treatment, radiotherapy, and chemotherapy [3]. The standard treatment for hepatocellular carcinoma (HCC) is surgical resection, combined with local radiotherapy and adjuvant chemotherapy with sorafenib. With long-term use, sorafenib has additional issues, such as toxicity and inefficacy. Therefore, it is important to find a novel and effective anticancer drug for the treatment of liver cancer as soon as possible. Certain compounds are cytotoxic to cancer cells, but normal cells are not affected [4].

**Scheme 1:**
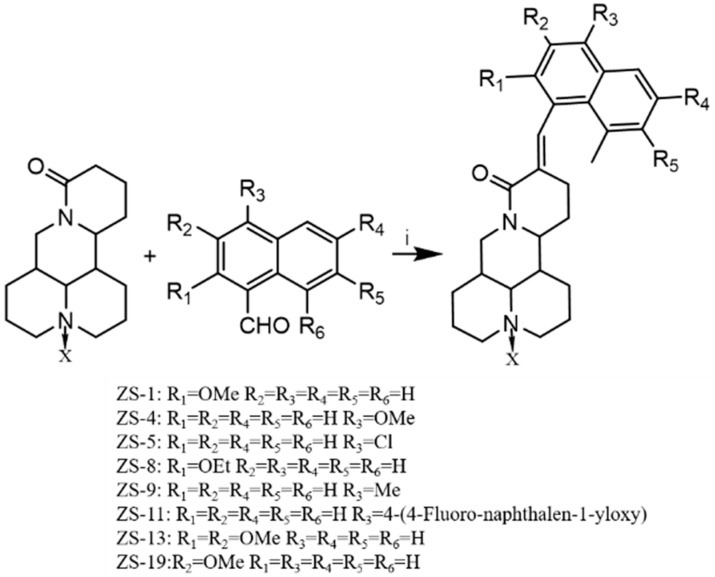
Synthetic route of matrine derivatives. Reaction conditions: (i) NaH, dry THF, reflux, 48 h.

Matrine (Scheme 1) is an alkaloid obtained from the dry roots of *Sophora flavescens* [5]. In vitro studies have shown that matrine possesses a wide range of pharmacological effects, such as anti-cancer, anti-inflammatory, and anti-bacterial activity [6,7]. Studies have shown that the pharmacological effects of matrine involve PI3K/Akt/mTOR, MAPK, and other signalling pathways and are closely related to apoptosis and immune regulation [8,9,10]. *S. flavescens* injection, a CFDA-approved anti-cancer drug, can be combined with other anti-tumour drugs to treat non-small cell lung cancer and liver cancer [11,12]. Matrine is the main ingredient of *S. flavescens*. Matrine is considered an ideal lead compound for further structural modification and optimisation because of its good solubility, safety, and flexibility. However, matrine exerts only moderate bioactivity and certain toxic effects, which limit its further application. Recently, a series of matrine derivatives have been synthesised. Studies have shown that these new derivatives exhibit significant effects in inhibiting tumour cell proliferation, invasion, and metastasis [13,14,15]. In a previous study, we found that modification of the 14 positions of the matrine skeleton could significantly improve its anti-tumour effect [16]. A series of new compounds were designed and synthesised in our laboratory.

Reactive oxygen species (ROS) are unstable molecules containing oxygen. The most common types of ROS include singlet hydrogen peroxide (H_2_O_2_), hydroxyl radicals (OH), and superoxide (O^2−^) [17]. Basal levels of ROS serve as physiological regulators of normal cell proliferation and differentiation, but large amounts of ROS can produce lipid peroxidation in biological membranes, leading to loss of membrane fluidity, abnormal membrane potential, rupture, and leakage of cellular content. ROS cause cell death through DNA damage-induced cell cycle arrest [18,19]. JNK is a stress-activated protein kinase associated with apoptosis and autophagy [20,21]. ROS-induced oxidative stress can activate underlying MAPK pathways [22]. In mammals, there are three JNK genes, *JNK1, JNK2*, and *JNK3* [23]. Studies have shown that ROS are involved in the classical apoptotic pathway and in activation of mitochondrial damage through proteins associated with mitochondrial function or cell death. [24,25].

In this study, we synthesised matrine derivatives and demonstrated that **ZS17** inhibited the growth, migration, and invasion of HCC cells in vivo and in vitro by activating the ROS-JNK-P53 pathway. The ROS-JNK-P53 pathway is involved in mitochondrial dysfunction in liver cancer cells. Thus, **ZS17** may represent a promising agent for the treatment of liver tumours in HCC patients. 

## 2. Results

### 2.1. MTT

**Scheme 2:**
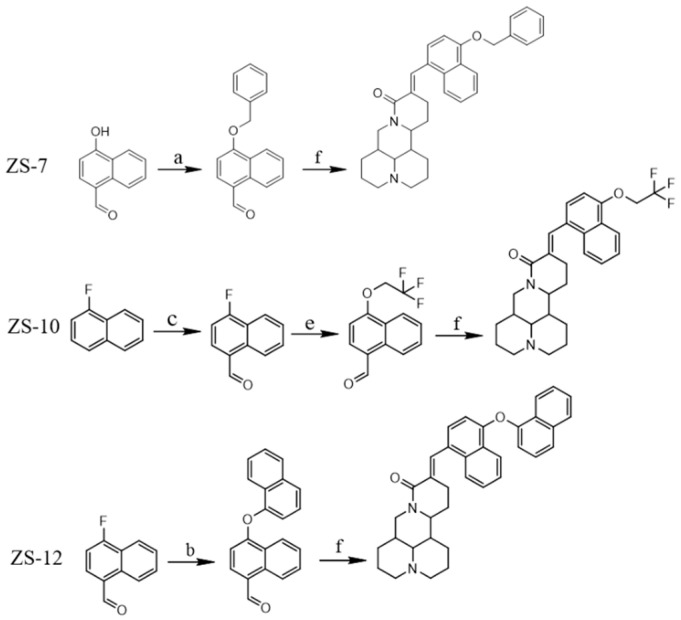
(**a**) K_2_CO_3_, benzyl bromide, acetone, rt, 24 h; (**b**) 1-Naphthol, 2-tert-Butyl-1,1,3,3-tetramethylguanidine, acetonitrile, 70 °C, 1 h; (**c**) Cl_2_CHOCH_3_, Sncl_4_, DCM, rt; (**e**): 2,2,2-Trifluoroethanol, DMSO, NaH, 70 °C; (**f**) matrine, NaH, Dry THF, 80 °C.

**Scheme 3:**
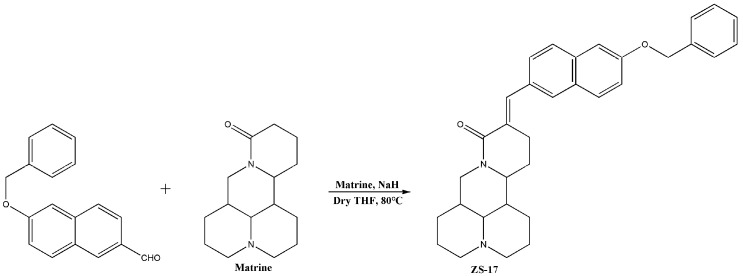
Synthetic route of matrine derivative **ZS17**.

**Figure 1:**
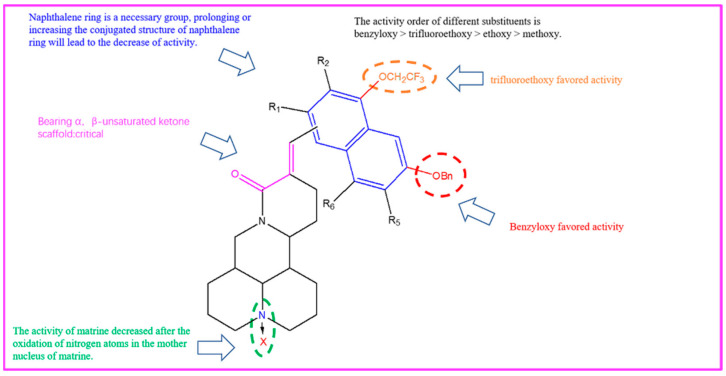
Structure–activity relationship for matrine derivatives.

Thirteen matrine derivatives (Scheme 1, Scheme 2 and Scheme 3) were designed and synthesised in our laboratory, and their toxicity to tumour cells was assayed using the MTT assay. To study the anti-tumour effects of the compounds in different tumours, four tumour cell lines were selected for the study. (Table 1) Among the 12 compounds, 9 displayed better antiproliferative activities with IC_50_ < 10 μM and 4 compounds with IC50 < 5 μM. Sorafenib was used as a positive control. The structure-activity relationship of sophoridine is shown in Figure 1. The compounds exhibited significant anti-tumour activity against different tumour cell lines. **ZS17** showed broad antitumour toxicity against four tumour cell types.

**Figure 2:**
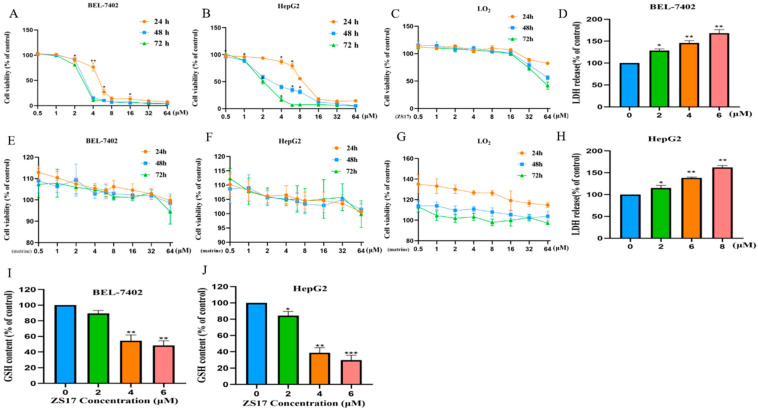
(**A**–**C**) Liver cancer cells (BEL-7402 and HepG2) and normal human liver cells (LO2) were treated with **ZS17** (0, 2, 4, 6 μM) for 24, 48, and 72 h. Cell viability was measured by MTT assay. (**E**–**G**) Liver cancer cells and normal human liver cells were treated with matrine. (**D**,**H**) Cells were treated with **ZS17** at a series of concentrations for 48 h. Cell cytotoxicity was evaluated using an LDH assay. (**I**,**J**) Percentage of GSH in cells after incubation with different concentrations of **ZS17** for 48 h. Data are expressed as mean ± SD. Compared with model group: * *p* < 0.5, ** *p* < 0.01, *** *p* < 0.001. n = 3 per group.

The inhibitory effects of **ZS17** on liver cancer cell lines were evaluated at increasing concentrations for 24, 48, or 72 h. The inhibitory activity (IC_50_) of **ZS17** against the growth of BEL-7402 and HepG2 cells was approximately 3.014 and 3.388 μM, respectively, after 48 h (Figure 2A–C). **ZS17** had a significant inhibitory effect on the proliferation of liver cancer cells compared with matrine exposure (Figure 2E–G). This suggested that **ZS17** selectively inhibits BEL-7402 and HepG2 cells. Lactate dehydrogenase is mainly located in the cytoplasm and can be used to reflect the destruction of cell membrane integrity in extracellular culture medium. The results showed that the release of LDH from BEL-7402 and HepG2 cells treated with different concentrations of **ZS17** for 48 h was dose-dependent (Figure 2D,H). Moreover, the nuclei were of different sizes, indicating that apoptosis of BEL-7402 and HepG2 cells was promoted by **ZS17**.

### 2.2. Migration and Invasion of BEL-7402 and HepG2 Cells Blocked by **ZS17**

**Figure 3:**
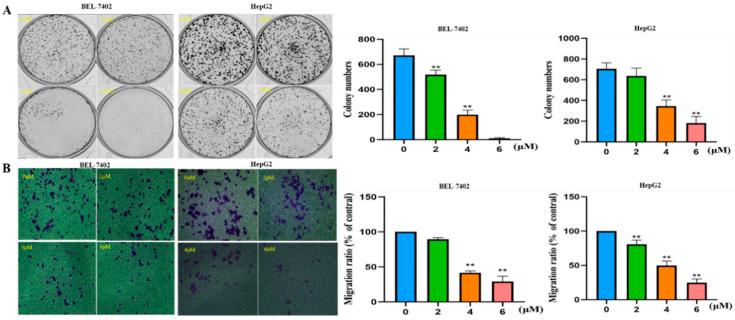
Suppression of cell lines grown in vitro. (**A**) Number of cell clones after treatment with **ZS17**. (**B**) Treatment with **ZS17** reduced the number of migrated cells in the transwell assay. Data are expressed as mean ± SD. Microscope’s magnification is 20×. Compared with model group: ** *p* < 0.01.

The results of colony formation assays showed that the number and size of colonies notably declined with increasing concentrations of **ZS17** (Figure 3A). The results of the invasion assay showed that the invasive ability of both BEL-7402 and HepG2 cells was inhibited when the concentration of **ZS17** reached 4 μM, indicating that **ZS17** had a significant effect on the invasive capacity of HCC cells.

### 2.3. Migration of BEL-7402 and HepG2 Cells Blocked by **ZS17**

**Figure 4:**
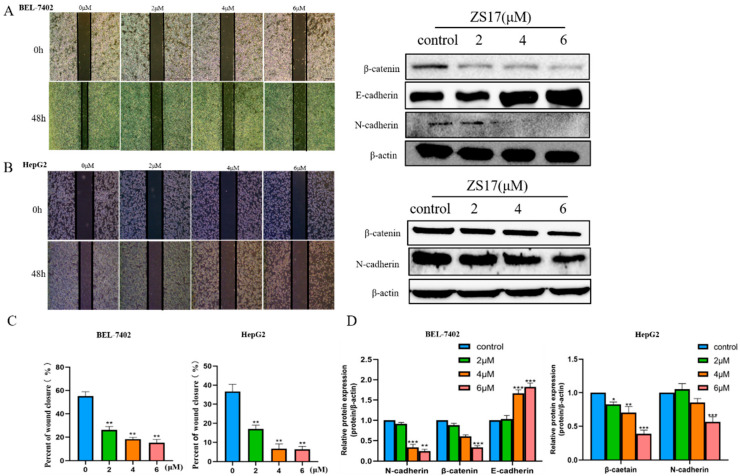
**ZS17** inhibits cell proliferation. (**A**,**B**) The wound-healing assay was used to estimate the migration. (**C**) Wound-healing rate of cells induced by **ZS17**. (**D**) Western blot analysis of the related proteins. Microscope’s magnification is 4×. Data are expressed as mean ± SD. Compared with model group: * *p* < 0.5, ** *p* < 0.01, *** *p* < 0.001. n = 3 per group.

The distance to wound closure was significantly shorter in the untreated group after 48 h (Figure 4A,B). When the concentration of **ZS17** was increased to 2 μM, the wound healing rates of BEL-7402 and HepG2 cells were approximately half that of the control group. **ZS17** significantly inhibited the migration of HCC cells during wound healing. Moreover, we observed an increased ratio of β-catenin to E-cadherin. N-cadherin expression was up-regulated after treatment with **ZS17**.

### 2.4. **ZS17** Induces Cell S-Phase Arrest and Apoptosis in BEL-7402 and HepG2 Cells

**Figure 5:**
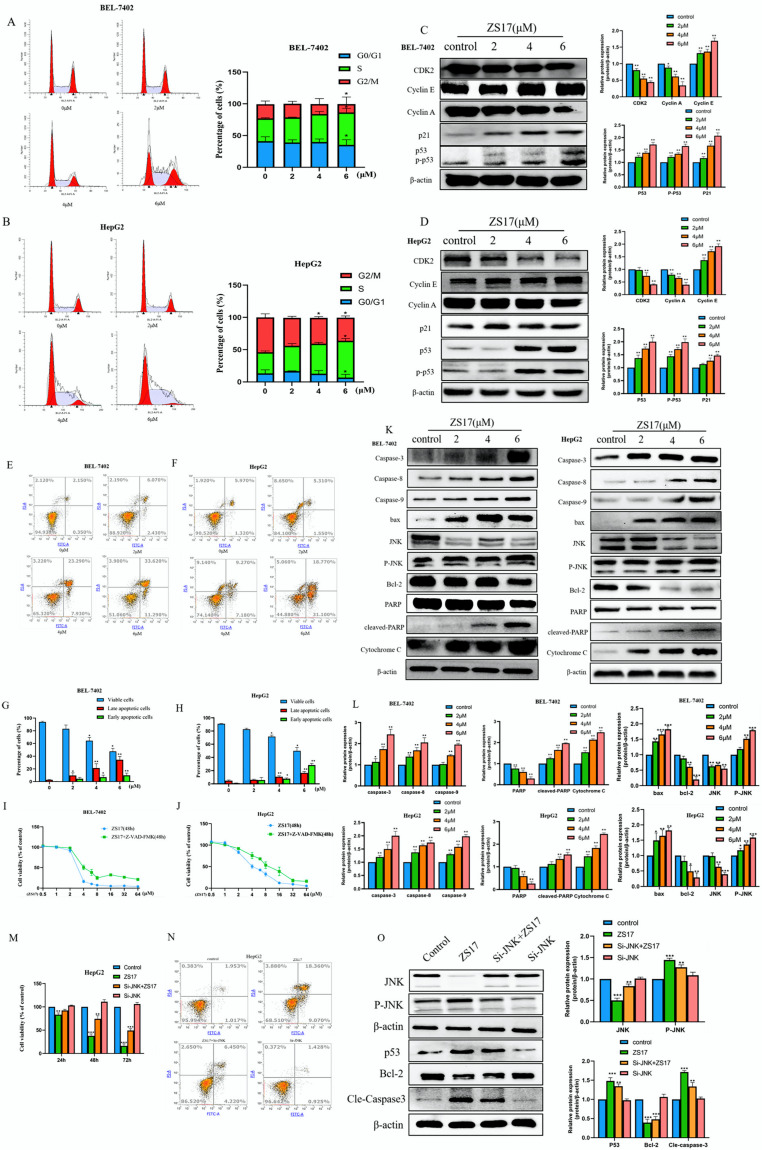
**ZS17** inhibited cell cycle arrest in S phase and induced liver cancer cell apoptosis. (**A**,**B**) Flow cytometry was used to determine the induction of cell cycle arrest in BEL-7402 and HepG2 cells after treatment with **ZS17** for 48 h. (**C**,**D**) Western blot analysis of related proteins. (**E**–**H**) Flow cytometry was used to detect the induction of apoptosis in BEL-7402 and HepG2 cells after treatment with **ZS17**. (**I**,**J**) Cell viability after treatment with **ZS17** and Z-VAD-FMK. (**K**,**L**) Western blot analysis of related proteins. (**M**) **ZS17** (4 μM) was applied to HepG2 cells transfected with YAP siRNA for 24 h, 48 h, and 72 h. (**N**) Detection of apoptosis in HepG2 cells after treatment with Si-JNK, **ZS17**, and Si-JNK + **ZS17**. (**O**) Detection of JNK, P-JNK, p53, Bcl-2, and Cle-Caspase protein expression levels in HepG2 cells by western blotting. Data are expressed as mean ± SD. Compared with model group: * *p* < 0.5, ** *p* < 0.01, *** *p* < 0.001. n = 3 per group.

After treatment with **ZS17** (4 μM and 6 μM), the number of S-phase cells increased significantly and the number of G2/M-phase cells decreased significantly, whereas the number of G0/G1-phase cells did not change significantly (Figure 5A,B). **ZS17** interrupts cell cycle progression by inducing S-phase arrest. We evaluated the effect of **ZS17** on apoptosis in liver cancer cells using annexin V-FITC/PI staining. Higher concentrations resulted in increased percentages of apoptotic cells compared to the control group in BEL-7402 and HepG2 **cells** (Figure 5E,F).

Z-VAD-FMK is a pan-caspase inhibitor that inhibits the activity of caspase family proteases. We further investigated whether **ZS17**-induced apoptosis was caspase-dependent. Z-VAD-FMK inhibited **ZS17**-induced apoptosis and increased the viability of **ZS17**-treated cells (Figure 5I,J).

Moreover, we found an increased ratio of caspase-3, 8, 9, P-JNK, Bax/Bcl-2, and PARP, and cleaved-PARP and JNK were upregulated after **ZS17** treatment. These results showed that **ZS17** treatment decreased the levels of CDK2, cyclin A, cyclin E, p21, and p53 in BEL-7402 and HepG2 cells (Figure 5C,D,K).

Knockdown of JNK by siRNA attenuated **ZS17**-induced p53 expression, increased the expression of the anti-apoptotic protein Bcl-2, and decreased the expression of cleaved caspase 3 (Figure 5M). Thus, it was shown that downregulation of JNK in HepG2 cells promoted cell proliferation and partially reversed the apoptosis-inducing effect of **ZS17** on HCC cells (Figure 5N,O).

### 2.5. **ZS17** Induces Liver Cancer Apoptotic Death by Evoking ROS Generation and Mitochondrial Dysfunction

**Figure 6:**
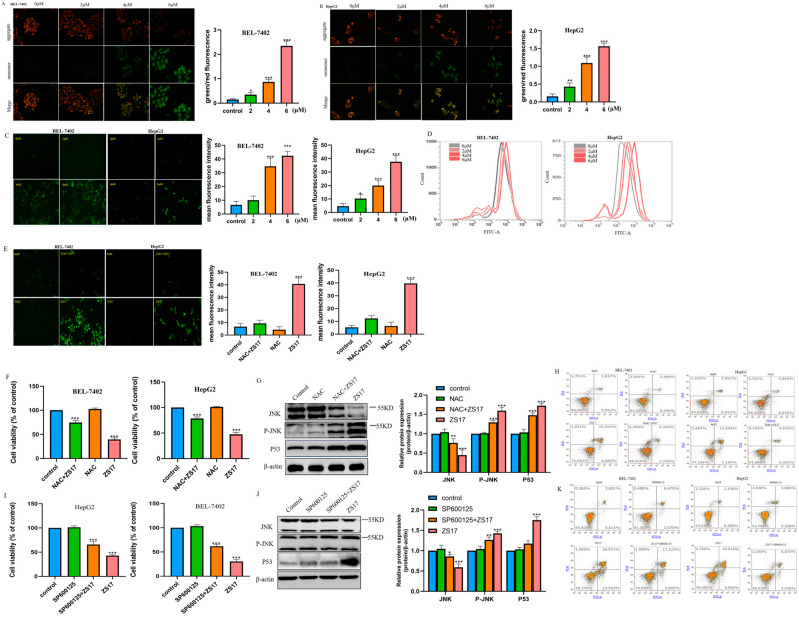
**ZS17** induces ROS generation and NAC inhibits ROS generation, attenuating **ZS17**-mediated apoptosis. (**A**,**B**) The mitochondrial membrane potential (MMP) was detected by a fluorescence microscope using JC-1 probe staining. (**C**) ROS generation was observed by fluorescence microscopy using a DCFH-DA probe. (**D**) ROS detection with DCFH-DA dye in different groups by flow cytometry. (**E**) BEL-7402 and HepG2 cells were pre-treated with NAC (5 mM, 1 h) and post-treated with **ZS17** (5 mM) for 24 h, and ROS generation was observed by fluorescence microscopy using DCFH-DA probe staining. (**F**,**I**) Cell proliferation ability was detected by MTT assay. (**G**) The expression of JNK and p-JNK in HepG2 cells treated with NAC, **ZS17**, and NAC + **ZS17** were detected by western blot. (**J**) The expression of JNK and p-JNK in HepG2 cells treated with NAC, **ZS17**, and NAC + **ZS17** was detected by western blot. (**H**,**K**) Apoptosis was detected by AnnexinV-FITC/PI staining and flow cytometry. Microscope’s magnification is 40×. Data are expressed as mean ± SD. Compared with model group: * *p* < 0.5, ** *p* < 0.01, *** *p* < 0.001. n = 3 per group.

It is widely known that mitochondria are the principal source of ROS in eukaryotic cells. Excessive ROS production can lead to DNA damage, mitochondrial dysfunction, and apoptosis. We explored the effect of **ZS17** on ROS generation in BEL-7402 and HepG2 cells, where green fluorescence of the DCFH-DA probe indicated intracellular ROS generation. The results showed that treatment of BEL-7402 and HepG2 cells with **ZS17** increased ROS generation in a dose-dependent manner (Figure 6C,D). Fluorescence intensity was higher in the treatment group than in the control group. **ZS17** (6 μM) exhibited the highest fluorescence intensity when applied to cells.

Significant changes in mitochondrial membrane potential were observed after 24 h of **ZS17** treatment in BEL-7402 and HepG2 cells (Figure 6A,B). The ratio of red fluorescence intensity to green fluorescence intensity decreased gradually in a concentration-dependent manner, suggesting destruction of the mitochondrial membrane and damage to the mitochondrial membrane. These results suggested that mitochondrial dysfunction may be involved in the induction of apoptosis in BEL-7402 and HepG2 cells after exposure to **ZS17**. Treatment of BEL-7402 and HepG2 cells with **ZS17** increased ROS generation in a dose-dependent manner (Figure 6C,D).

ROS levels produced by BEL-7402 and HepG2 cells were measured after treatment with **ZS17** and pretreatment with 5 mM NAC for 1 h. ROS increased with increasing concentrations of **ZS17**, whereas the green fluorescence of the DCFH-DA probe was significantly decreased in the NAC pretreatment group (Figure 6E). The viability of cells pretreated with NAC was higher than that of cells treated with **ZS17** alone (Figure 6F). NAC prevented **ZS17**-induced JNK and p-JNK expression, indicating that **ZS17**-induced ROS generation was responsible for JNK activation and mediation of anti-tumour activities in liver cancer cells (Figure 6G). Our results suggested that accumulation of intracellular ROS leads to **ZS17**-induced apoptosis. To further investigate whether ROS are involved in **ZS17**-induced apoptosis, cells were treated with NAC, which significantly reduced the rate of **ZS17**-induced apoptosis (Figure 6H).

JNK activation plays a crucial role in the apoptosis of many cancer cells by releasing the pro-apoptotic protein Bax, which induces apoptosis [26,27]. Pretreatment of HCC cells with the JNK inhibitor SP600125 demonstrated the importance of JNK in **ZS1**7-mediated anti-tumour activity (Figure 6J). Our results showed that SP600125 significantly attenuated the antiproliferative (Figure 6I) and apoptotic effects (Figure 6K) of **ZS17** in BEL-7402 and HepG2 cells. **ZS17**-induced apoptosis in HCC cells was partially mediated through the ROS-JNK-P53 signalling pathway.

### 2.6. ROS Is Involved in **ZS17**-Induced Mitochondrial Dysfunction

**Figure 7:**
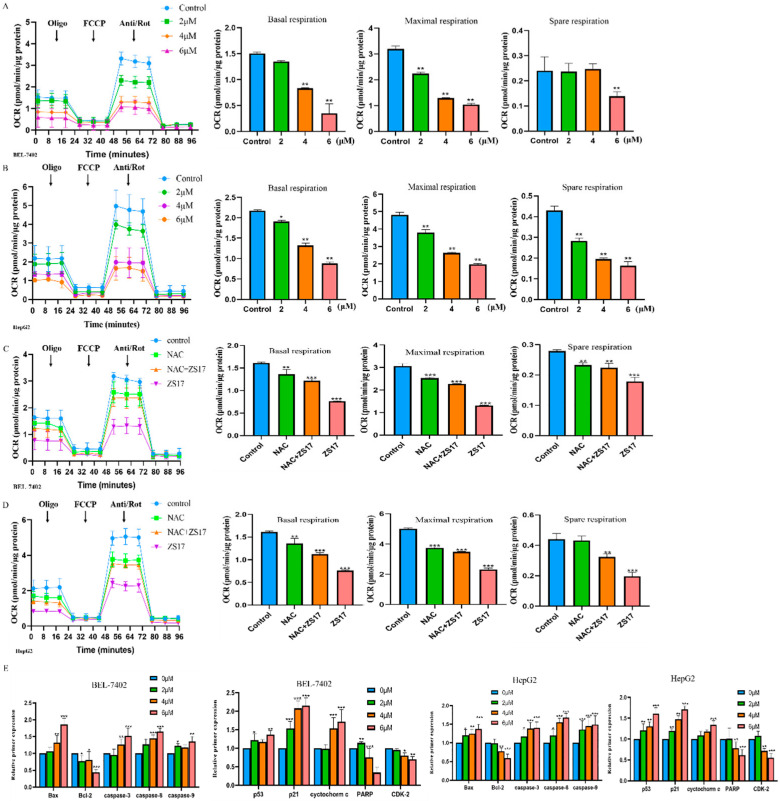
(**A**–**D**) Cellular oxygen consumption rate (OCR) was measured in real-time using the Seahorse XF96 Extracellular Flux Analyzer (**C**,**D**) **ZS17** and co-treated with NAC in BEL-7402 and HepG2 cells. (**E**) The expression of relevant proteins at the mRNA level was detected by q-PCR. Data are expressed as mean ± SD. Compared with model group: * p < 0.5, ** p < 0.01, *** p < 0.001. n = 3 per group.

To investigate the effects of **ZS17** on mitochondrial respiration, real-time oxidative phosphorylation was assessed by measuring cellular OCR (oxygen consumption rate). The results revealed that **ZS17** markedly decreased basal respiration, maximal respiration, spare respiration, and ATP production in BEL-702 and HepG2 cells (Figure 7A,B). These results suggested that **ZS17** inhibits cellular mitochondrial respiration. Furthermore, the combined treatment of BEL-7402 and HepG2 cells with the ROS inhibitor NAC and **ZS17** (4 μM) significantly attenuated the inhibitory effects of **ZS17** on OCR as well as basal, maximal, and alternate respiration (Figure 7C,D). This study suggested that inhibition of mitochondrial respiration in hepatoma cells by **ZS17** is mediated by ROS production. Research suggests that mitochondrial metabolism is an attractive target for tumour therapy [28,29]. **ZS17** activates the ROS-JNK-p53 pathway, inhibits mitochondrial oxidative phosphorylation, induces apoptosis, and may further lead to mitochondrial dysfunction in tumour cells by inhibiting ATP production by HCC cells.

Moreover, **ZS17** increased the mRNA expression levels of *Bax*, caspase-3, caspase-8, caspase-9, *p21*, *p53*, and cytochrome c (*CYCS*) in BEL-7402 and HepG2 cells and decreased the mRNA expression levels of *JNK, Bcl-2, PARP*, and *CDK-2* (Figure 7E).

### 2.7. Anti-Tumour Activity and Toxicity Assessment of **ZS17** in Zebrafish In Vivo

**Figure 8:**
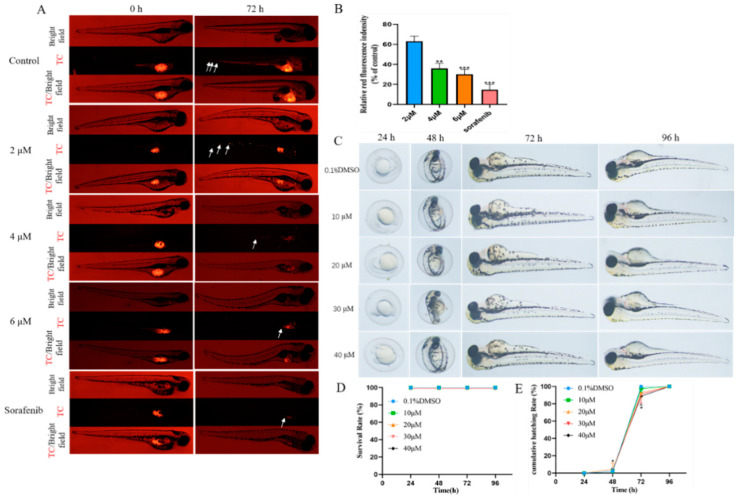
Inhibitory effect of **ZS17** on the proliferation of HepG2 cells in a zebrafish xenograft model and toxicity assessment of **ZS17**. (**A**) Proliferation of HepG2 cells in zebrafish after treatment with **ZS17** (0, 2, 4, and 6 μM). White arrows: tumor cells. (**B**) Quantified relative red fluorescence in zebrafish embryos using ImageJ software. (**C**) Ecotoxicology study of different concentrations. (**D**,**E**) Survival rate and cumulative hatching rate of zebrafish embryos treated with **ZS17**. Microscope’s magnification is 40×. Data are expressed as mean ± SD. Compared with model group: ** *p* < 0.01, *** *p* < 0.001. n = 3 per group.

The zebrafish xenograft model is an important model for in vivo studies. HepG2 cells with red fluorescence were injected into the zebrafish yolk gap. Zebrafish were cultured with **ZS17** (0, 2, 4, and 6 μM). The results showed that after 72 h, the red fluorescence intensity and fluorescence area of **ZS17**-treated HepG2 cells were significantly lower than those of a control group (Figure 8A). In addition, red fluorescence was quantified using ImageJ software (Figure 8B). After treatment of HepG2 cells with 6 μM **ZS17** for 72 h, the proliferation rate was lower than that of cells without **ZS17** and with 2 μM **ZS17**. Experimental results showed that **ZS17** effectively blocked the proliferation and metastasis of cancer cells in vivo.

#### Toxicity Evaluation of **ZS17** Using Zebrafish Embryos

Toxicity assessment determinations indicated that the entire zebrafish embryo hatched normally without any defects when **the ZS17** concentration reached 40 μM. Moreover, none of the zebrafish exhibited any significant developmental abnormalities compared with the control group, and there were no cases of acute death. These results further suggested that **ZS17** has non-significant developmental toxicity.

### 2.8. In Vivo Anti-Liver Tumour Activity of **ZS17**

**Figure 9:**
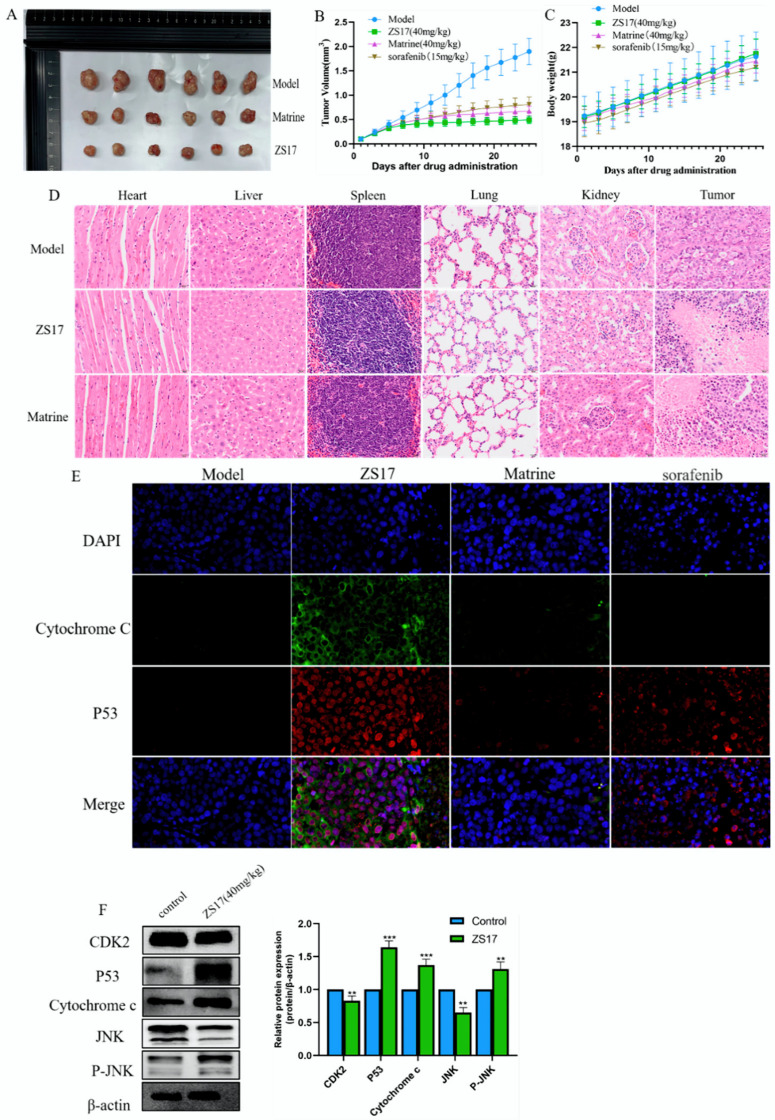
**ZS17** inhibited tumor growth in human HepG2 xenograft nude mouse model. (**A**,**B**) Tumor growth curves and volume in **ZS17**-treatment and model groups. (**C**) Body weight curves. (**D**) Histopathological analysis of mouse heart, livers, spleen, lung, kidneys, and tumor by hematoxylin and eosin (H&E) staining. (**E**) IF expression of Cytochrome c and p53 proteins in tumor tissue. (**F**) Western blot assays using lysates of isolated tumors and indicated antibodies. There were six nude mice in each group. Microscope’s magnification is 40×. Data are expressed as mean ± SD. Compared with model group: ** *p* < 0.01, *** *p* < 0.001.

Tumours were found in the dissected liver tissue of the vehicle and matrine groups, whereas tumour sizes decreased dramatically in **the ZS17** (40 mg/kg) group (Figure 9A,B). Moreover, **ZS17** treatment did not lead to a reduction in body weight (Figure 9C). H&E staining showed that **ZS17** was not significantly toxic to the major organs of nude mice, including the heart, liver, spleen, lungs, and kidneys (Figure 9D). These results demonstrated that **ZS17** significantly suppressed tumour growth with no obvious side effects, whereas matrine did not exhibit any noticeable antitumour activity. Immunofluorescence analyses showed that expression of P53 and cytochrome c was increased in mouse tumour tissues after treatment of mice with **ZS17** (Figure 9E). As expected, **ZS17** showed more potent effects than matrine did. Western blotting results revealed that **ZS17** downregulated JNK and CDK2 and upregulated P-JNK, p53, and cytochrome c in vivo (Figure 9F).

## 3. Discussion

Matrine is an alkaloid isolated from *S. flavescens* and has been widely used in China for many years as an adjuvant treatment for liver, lung, and stomach cancers, with few side effects. [30,31]. Accumulated ROS production in cancer cells has been reported to inhibit cell proliferation and induce DNA and cellular damage. A large increase in ROS production can lead to the apoptosis of cancer cells. Increasing the production of ROS in cancer cells without causing significant toxicity in normal cells may be a potential therapeutic approach. Studies have shown that increased ROS production can activate the JNK signalling pathway and lead to apoptosis [32]. In this study, 12 new matrine derivatives were synthesised, among which **ZS17** effectively inhibited the proliferation and migration of liver cancer cells in vitro and in vivo.

Apoptosis is a physiological process that regulates cell death in response to diseases and exogenous stress [33,34]. Induction of apoptosis is an efficient strategy for cancer treatment [35]. ROS are highly reactive forms of molecular oxygen and basal levels of ROS are physiological regulators of normal proliferation and differentiation. Evidence suggests that JNK is primarily activated by various environmental stressors including oxidative stress [36]. Previous studies have shown that activation of the JNK pathway induces mitochondria-dependent apoptosis through activation of mitochondrial Bcl-2 family proteins and caspase-3 [37].

Our studies showed that stimulation of BEL-7402 and HepG2 cells by **ZS17** leads to activation and phosphorylation of JNK, which induces the accumulation of p53. Activated p53 in turn may enhance JNK signalling through a positive feedback loop between p53 and JNK. JNK phosphorylates p53 (Ser 15), which is activated in its N-terminal activation loop [38]. Activated p53 leads to a decrease in the mitochondrial transmembrane potential (JM) and the release of cytochrome c from mitochondria into the cytoplasm, where it further forms an apoptotic complex. This apoptotic complex, consisting of APAF-1, caspase-9, and cytochrome c, damages mitochondrial DNA and induces apoptosis [39]. Mitochondrial apoptosis is mainly regulated by the Bcl-2 family of proteins including Bcl-2 and Bax [40].

**Figure 10:**
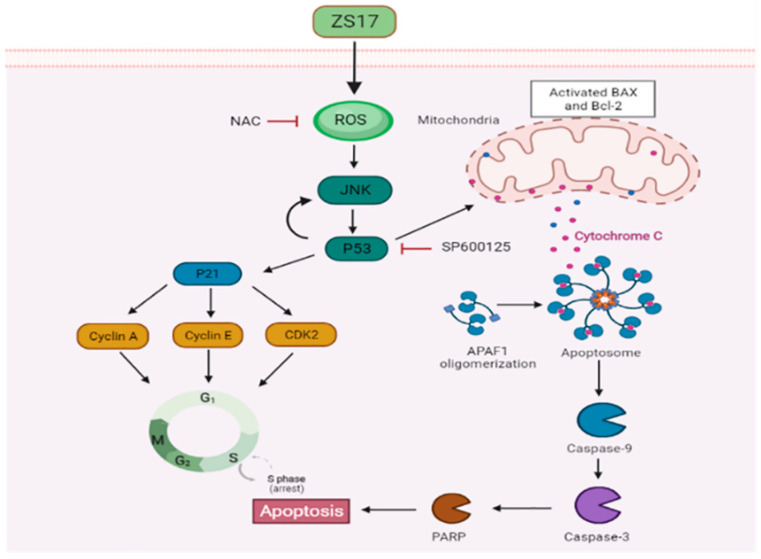
Inhibition mechanism of liver tumor growth by **ZS17**.

When Bcl-2 protein expression decreases [41], Bax is transferred to the mitochondrial membrane, its expression increases and the ratio of Bax/Bcl-2 changes, which stimulates the release of proapoptotic factors from the mitochondria. It also activates the caspase cascade and inhibits PARP protein expression in a concentration-dependent manner, ultimately inducing apoptosis via the mitochondrial pathway. Thus, elevated ROS levels can further induce apoptosis in HCC cells via the ROS-JNK-P53 pathway. Activation of the tumour suppressor protein p53 can lead to cell cycle arrest and apoptosis. Accumulation of p53 in the nucleus (Figure 4C,D and Figure 8E) led to cell cycle arrest. p21 expression is upregulated by p53 phosphorylation. p21 protein is an inhibitor of cell cycle progression and CDKS [42,43], which binds to CDK2 to form a complex that inhibits kinase activity and blocks progression into the G1/S phase. Cyclin A is required for both G1- and S-phase DNA initiation and elongation, whereas p21 inhibits cyclin A and cyclin E, thereby preventing transfer of the cell cycle from the S-phase to the G2/M phase, resulting in S-phase cell cycle arrest. The potential mechanism of apoptosis induced by ZS17 is shown in Figure 10.

GSH is a natural antioxidant that scavenges free radicals such as ROS. Our results showed that ZS17 reduced cellular GSH levels, further reducing the antioxidant capacity of cells. The ROS inhibitor NAC was validated, and NAC effectively blocked ZS17-induced apoptosis. ZS17 likely induces oxidative damage in BEL-7402 and HepG2 cells via ROS generation to trigger cell apoptosis. In addition, Z-VAD-FMK pretreatment almost completely reversed ZS17-induced apoptosis. SP600125, a specific inhibitor of JNK, inhibited the induction of apoptosis by ZS17, suggesting that the JNK signalling pathway mediates the apoptotic effects of ZS17 in cells. In addition, inhibition of ROS production by NAC significantly increased cell survival and inhibited apoptosis. In addition to cellular effects, our study demonstrated efficient inhibition of tumour growth by ZS17 in a nude mouse tumour model (Figure 8). Moreover, it effectively inhibited tumour cell migration in a zebrafish tumour model (Figure 7). ZS17 also exhibited a high level of safety in both mice and zebrafish (Figure 8). These results suggested that ZS17 induces apoptosis by inducing ROS-mediated oxidative damage.

## 4. Materials and Methods

### 4.1. General Experimental Procedures

MCF-7, SGC-7901, H460, BEL-7402, and HepG2 cell lines were obtained from the Type Culture Collection of the Chinese Academy of Sciences (Shanghai, China). BEL-7402 was grown in RPMI 1640 (Gibco, China) supplemented with 10% fetal bovine serum (FBS; Gibco/Thermo Fisher Scientific, Waltham, MA, USA). HepG2 was grown in DMEM media (Gibco/Thermo Fisher Scientific) supplemented with 10% FBS. All cells were maintained at 37 °C in a humidified incubator containing 5% CO2. Female BALB/c nude mice (5–6 weeks) purchased from Vital River (SCXK (Jing) 2016-0006, Beijing, China) and AB-type zebrafish purchased from the National Zebrafish Resource Centre, (Beijing, China) were used for the in vivo experiments. Oligomycin, FCCP, and rotenone were purchased from Sigma (St. Louis, USA). Matrine were purchased from Angsheng Biological medicine (Shanxi, China, 98.6%). The Si-YAP were transfected into HepG2 cells with lipofectamine 3000. Cells were incubated for 48 h, and the expression of JNK was detected by western blot. JNK, 5′- GCCGACCAUUUCAGAAUCATT -3′.

### 4.2. Antibodies and Reagents

The following antibodies were used: p53(#2524T), p-p53(#82530), p21(#2947T), PARP(#9542T), C-PARP(#5625), CDK2(#2546T), cyclin E(#20808T), Cyclin A(#4656T), Cytochrome c(#4280T), caspase-3(#9662T), Cle-caspase-3(#9664), caspase-8(#4790T), caspase-9(#9508T), N-cadherin(#13116), E-cadherin(#14472), β-catenin(#8480), β-actin(#4970T), JNK(#9252), Phospho-SAPK/JNK Thr183/Tyr185(#4668), anti-Rabbit IgG, and anti-mouse IgG; all were purchased from Cell Signaling Technology (CST, Danvers, MA, USA); Bax (Cat# ab77566) and Bcl-2 (Cat# ab59348) were obtained Abcam (1:1000). 

Other reagents used in the present study were: MTT, N-acetylcysteine (NAC; ROS scavenger), SP600125, and Z-VAD-FMK purchased from MedChemExpress (Monmouth Junction, NJ, USA). Antibody diluent and Restore™ PLUS Western Blot Stripping Buffer were purchased from Beyotime (Nanjing, China). Lactate dehydrogenase (LDH), 4’,6-diamidino-2-phenylindole (DAPI), 2,7-dichlorofluorescein diacetate (DCFH-DA), mitochondrial membrane potential assay kits with JC-1 were purchased from Beyotime (Nanjing, China). Cell cycle and apoptosis measurement kit were purchased from Becton, Dickinson, and Company. The primers used for the amplification of β-actin, p53, p21, Cyctochorm C, Bax, Bcl-2, Caspase-3, Caspase-8, Caspase-9, PARP, Fas, JNK, MMP2, MMP9, and CDK-2 were synthesized by Generay Co., Ltd. (Shanghai, China). β-actin sequence: forward primer (5’-3), TCAAGAAGGTGGTGAAGCAG; reverse primer (5’-3’), TCGCTGTTGAAGTCAGAGGA. p53 sequence: forward primer (5’-3), CAGCACATGACGGAGGTGGT; reverse primer (5’-3’), TCATCCAAATACTCCACACGC. p21 sequence: forward primer (5’-3’), TGTCCGTCAGAACCCATGC; reverse primer (5’-3’), AAAGTCGAAGTTCCATCGCTC. Cyctochorm c sequence: forward primer (5’-3’), CACCGTTGAAAAGGGAGGC; reverse primer (5’-3’), TGTTCTTATTGGCGGCTGT. Bax sequence: forward primer (5’-3’), CCCGAGAGGTCTTTTTCCGAG; reverse primer (5’-3’), CCAGCCCATGATGGTTCTGAT. Bcl-2 sequence: forward primer (5’-3’), GGTGGGGTCATGTGTGTGG; reverse primer (5’-3’), CGGTTCAGGTACTCAGTCATCC. Caspase-3 sequence: forward primer (5’-3’), CATGGAAGCGAATCAATGGACT; reverse primer (5’-3’), CTGTACCAGACCGAGATGTCA. Caspase-8 sequence: forward primer (5’-3’), TTTCTGCCTACAGGGTCATGC; reverse primer (5’-3’), TGTCCAACTTTCCTTCTCCCA. Caspase-9 sequence: forward primer (5’-3’), CTCAGACCAGAGATTCGCAAAC; reverse primer (5’-3’), GCATTTCCCCTCAAACTCTCAA. PARP sequence: forward primer (5’-3’), CGGAGTCTTCGGATAAGCTCT; reverse primer (5’-3’), TTTCCATCAAACATGGGCGAC. CDK-2 sequence: forward primer (5’-3’), CCAGGAGTTACTTCTATGCCTGA; reverse primer (5’-3’), TTCATCCAAGGGGAGGTACAAC. JNK sequence: forward primer (5’-3’), TGTGTGGAATCAAGCACCTTC; reverse primer (5’-3’), AGGCGTCATCATAAAACTCGTTC.

### 4.3. Chemistry

All chemicals used in this study were purchased from Energy Chemical (Shanghai, China) and were of analytical purity. (Scheme 1). The process was examined by thin-layer chromatography (TLC) under UV illumination. Nuclear magnetic resonance hydrogen (Bruker600, Berlin, Germany,) and carbon spectroscopy were used for structural identification.

Then, 116 mmol (2.8 g) of sodium hydride and 50.0 mL of anhydrous tetrahydrofuran were added to a 100 mL round bottom flask and stirred well. Then, 5 mmol (1.24 g) of matrine was added, and the temperature was slowly increased to 80 °C. Next, 10 mmol (1.86 g) of 2-methoxy-1-naphthaldehyde was added and reacted to the endpoint (TLC detection). The reaction solution was cooled and adjusted to neutral pH with 3 N hydrochloric acid. The organic phase was combined and dried over anhydrous Na_2_SO_4_, and filtered and concentrated to yield a yellow oil. Purification by silica gel column chromatography (eluent: V ethyl acetate: V petroleum ether = 5:4) resulted in a white solid, which was recrystallised from ethyl acetate–methanol to give 1.81 g of white crystals (**ZS-1**).

Preparation of compounds using a synthetic method for **ZS-1.** (Scheme 1) The intermediate products of the reactions were purchased from Energy Chemical and BidePharm (Shanghai, China).

#### 4.3.1. 14-[2-(6-Benzyloxy) Naphthylenyl] Matrine (**ZS17**)

Matrine (5 mmol, 1.24 g), sodium hydride (116 mmol, 2.8 g), 6-benzyloxy-2-naphthaldehyde (1.86 g, 10 mmol), and tetrahydrofuran (50 mL) were mixed and heated to 80 °C for 6 h. Then, 20 mL of water was added, and the solution pH was adjusted to 7 using HCl. The crude product was filtered and vacuum-dried, and a yellow precipitate was obtained. The sample was purified via column chromatography using a mixture of ethyl acetate and petroleum ether (5:4).

#### 4.3.2. 14-[1-(2-Methoxy) Naphtholenyl] Matrine (ZS-1)

White crystals, total yield: 83.0%, m.p.174.4~176.6 °C; purity by HPLC: 96%. ^1^H NMR (600 MHz, Chloroform-d) δ: 7.89(s, 1H), 7.84(d, J = 9.0 Hz, 1H), 7.82~7.78(m, 2H), 7.45(ddd, J = 8.4, 6.7, 1.3 Hz, 1H), 7.36(ddd, J = 7.9, 6.7, 1.1 Hz, 1H), 7.30(d, J = 9.0 Hz, 1H), 4.59(dd, J = 12.6, 4.4 Hz, 1H), 4.00~3.93(m, 1H), 3.92(s, 3H), 3.26(t, J = 12.6 Hz, 1H), 2.89~2.78(m, 2H), 2.32~2.25(m, 1H), 2.18~2.13(m, 2H), 2.08~1.96(m, 4H), 1.89~1.82(m, 2H), 1.82~1.75(m, 2H), 1.69~1.54(m, 3H), 1.52~1.45(m, 2H), 1.45~1.36(m, 2H); ^13^C NMR (151 MHz, Chloroform-d) δ: 164.55, 154.17, 134.05, 132.53, 129.46, 128.88, 128.76, 128.11, 126.49, 124.81, 123.65, 119.19, 113.37, 63.84, 57.30, 56.46, 53.24, 42.83, 42.51, 35.61, 27.85, 26.43, 25.78, 24.09, 21.25, 20.81; ESI-HRMS m/z:calcd for C_27_H_32_N_2_O_2_[M + 1]+ 417.2849, found 417.2820.

#### 4.3.3. 14-[1-(4-Methoxy) Naphtholenyl] Matrine (ZS-4)

White powder, total yield: 56.0%, m.p.183.5~184.2 °C; purity by HPLC: 96.3%. ^1^H NMR (600 MHz, Chloroform-d) δ: 8.31~8.26(m, 1H), 8.16(d, J = 1.6 Hz, 1H), 7.98~7.93(m, 1H), 7.53~7.46(m, 2H), 7.23(dd, J = 7.9, 1.0 Hz, 1H), 6.79(d, J = 8.0 Hz, 1H), 4.56(dd, J = 12.7, 4.4 Hz, 1H), 4.00(s, 3H), 3.98~3.91(m, 1H), 3.24(t, J = 12.7 Hz, 1H), 2.88~2.78(m, 2H), 2.72(dddd, J = 14.9, 7.0, 3.7, 1.2 Hz, 1H), 2.38(dddd, J = 14.9, 11.1, 3.8, 1.9 Hz, 1H), 2.12(d, J = 3.0 Hz, 1H), 2.04(s, 4H), 2.00~1.93(m, 2H), 1.86~1.81(m, 2H), 1.80~1.72(m, 2H), 1.68~1.51(m, 3H), 1.49~1.33(m, 3H); ^13^C NMR (151 MHz, Chloroform-d) δ: 164.96, 155.46, 132.93, 132.61, 131.60, 127.09, 126.68, 125.66, 125.50, 125.29, 124.73, 122.28, 103.03, 63.79, 57.24, 55.51, 52.98, 42.80, 42.64, 35.65, 27.80, 26.36, 26.26, 23.48, 21.19, 20.77; ESI-HRMS m/z:calcd for C_27_H_32_N_2_O_2_[M + 1]+ 417.2814, found 417.2820.

#### 4.3.4. 14-[1-(4-Cl) Naphtholenyl] Matrine (ZS-5)

Light yellow powder, total yield: 27.0%, m.p.175.3~178.1 °C; purity by HPLC: 98.1%. ^1^H NMR (600 MHz, Chloroform-d) δ: 8.32(dd, J = 8.5, 1.2 Hz, 1H), 8.18~8.13(m, 1H), 8.04~7.99(m, 1H), 7.65~7.61(m, 1H), 7.59~7.55(m, 2H), 7.22(dd, J = 7.7, 1.0 Hz, 1H), 4.58(ddd, J = 12.3, 6.7, 4.4 Hz, 2H), 3.98(ddd, J = 10.7, 8.3, 5.2 Hz, 2H), 3.27(t, J = 12.7 Hz, 2H), 2.92~2.77(m, 4H), 2.75~2.57(m, 2H), 2.36(ddddd, J = 14.9, 13.1, 11.2, 3.8, 2.0 Hz, 2H), 2.15(d, J = 3.0 Hz, 2H), 2.12~2.03(m, 2H), 1.99(tdd, J = 12.3, 6.8, 2.8 Hz, 3H), 1.89~1.82(m, 4H), 1.82~1.74(m, 3H), 1.68~1.54(m, 4H), 1.52~1.38(m, 6H); ESI-HRMS m/z:calcd for C_27_H_32_N_2_O_2_[M + 1]+ 421.226, found 421.2268.

#### 4.3.5. 14-[1-(4-Benzyloxy) Naphtholenyl] Matrine (ZS-7)

Next, 3.96 g (23.02 mmol) of 4-hydroxy-1-naphthaldehyde was dissolved in 60 mL of acetone and 3.5 g (25.32 mmol) of potassium carbonate and 5.91 g (34.53 mmol) of benzyl bromide were added with stirring. The reaction mixture was stirred at room temperature (25 °C) for 24 h and monitored using TLC. After the reaction was complete, 100 mL of ethyl acetate was added, and the product was filtered under reduced pressure. The filtrate was concentrated to obtain a brownish-red product. The product was purified by silica gel column chromatography (V ethyl acetate: V hexane = 1:20) to give 5.9 g a yellow oil with 98% yield. (4-Benzyloxy-1-naphthaldehyde).

Next, **ZS-7** was prepared by referring to the synthesis of ZS-1.

White powder, total yield: 49.0%, m.p.199.3~202.7 °C; purity by HPLC: 96.3%. ^1^H NMR (600 MHz, Chloroform-d) δ: 8.41(dd, J = 8.0, 1.6 Hz, 1H), 8.20(s, 1H), 8.04~7.97(m, 1H), 7.57~7.52(m, 4H), 7.46~7.41(m, 2H), 7.40~7.33(m, 1H), 7.24(d, J = 7.8 Hz, 1H), 6.88(dd, J = 8.0, 2.3 Hz, 1H), 5.26(d, J = 3.0 Hz, 2H), 4.59(dd, J = 12.7, 4.4 Hz, 1H), 3.97(ddd, J = 10.8, 8.2, 5.3 Hz, 1H), 3.27(t, J = 12.6 Hz, 1H), 2.89~2.79(m, 2H), 2.73(ddd, J = 15.1, 7.0, 3.7 Hz, 1H), 2.39(dddd, J = 14.9, 11.2, 3.8, 1.9 Hz, 1H), 2.13(t, J = 3.0 Hz, 1H), 2.11~2.03(m, 1H), 1.98(tdd, J = 12.0, 6.2, 2.6 Hz, 2H), 1.90~1.72(m, 4H), 1.69~1.53(m, 3H), 1.51~1.36(m, 4H); ^13^C NMR (151 MHz, Chloroform-d) δ: 164.90, 154.54, 136.96, 133.06, 132.47, 131.79, 128.63, 128.01, 127.37, 127.07, 126.78, 126.00, 125.71, 125.41, 124.80, 122.54, 104.46, 70.14, 63.78, 57.28, 53.02, 42.82, 42.67, 35.68, 27.85, 26.41, 26.26, 23.55, 21.26,20.83; 

#### 4.3.6. 14-[1-(2-Ethoxy) Naphtholenyl] Matrine (ZS-8)

Yellow powder, total yield: 42.0%, m.p.176.4~178.2 °C; purity by HPLC: 98.9%. ^1^H NMR (600 MHz, Chloroform-d) δ: 7.91(d, J = 10.2 Hz, 1H), 7.83~7.79(m, 3H), 7.45(ddd, J = 8.3, 6.7, 1.4 Hz, 1H), 7.37(ddd, J = 8.0, 6.7, 1.2 Hz, 1H), 7.30~7.27(m, 2H), 4.60(dd, J = 12.7, 4.4 Hz, 1H), 4.20~4.12(m, 3H), 4.02~3.91(m, 1H), 3.27(t, J = 12.7 Hz, 1H), 3.11~3.00(m, 1H), 2.86(dd, J = 22.7, 11.4 Hz, 2H), 2.35~2.22(m, 2H), 2.20~2.12(m, 2H), 2.08~1.96(m, 4H), 1.92~1.84(m, 2H), 1.83~1.75(m, 2H), 1.70~1.56(m, 3H), 1.52~1.47(m, 1H), 1.42(q, J = 6.8 Hz, 5H); ^13^C NMR (151 MHz, Chloroform-d) δ: 165.95, 158.37, 134.75, 133.73, 129.17, 128.68, 128.54, 128.11, 127.39, 124.81, 124.35, 119.49, 113.37, 65.0, 63.84, 57.30, 56.46, 53.24, 42.83, 42.51, 36.13, 27.35, 26.98, 25.47, 24.38, 21.05, 20.88; ESI-HRMS m/z: calcdfor C_28_H_34_N_2_O_2_[M + 1]+ 431.2934, found431.2956.

#### 4.3.7. 14-[1-(4-Methyl) Naphthalenyl] Matrine (ZS-9)

Yellow powder, total yield: 53.0%, m.p.214.4~21.6.2 °C; purity by HPLC: 97.9%. ^1^H NMR (600 MHz, Chloroform-d) δ: 8.19(s, 1H), 8.03~7.97(m, 2H), 7.50(dddd, J = 20.5, 8.1, 6.8, 1.4 Hz, 2H), 7.28(dd, J = 6.8, 1.2 Hz, 1H), 7.19(dd, J = 7.1, 0.9 Hz, 1H), 4.56(dd, J = 12.6, 4.4 Hz, 1H), 3.94(ddd, J = 10.8, 8.2, 5.2 Hz, 1H), 3.24(t, J = 12.6 Hz, 1H), 2.80(ddd, J = 23.3, 9.4, 3.5 Hz, 2H), 2.72~2.62(m, 4H), 2.34 (dddd, J = 14.9, 11.2, 3.8, 2.0 Hz, 1H), 2.10(t, J = 3.1 Hz, 1H), 2.03(s, 3H), 1.95(tdd, J = 11.8, 5.1, 2.1 Hz, 2H), 1.84~1.77(m, 2H), 1.77~1.71(m, 1H), 1.65~1.49(m, 3H), 1.49~1.32(m, 3H); ^13^C NMR (151 MHz, Chloroform-d) δ: 164.69, 134.52, 132.72, 132.56, 132.44, 131.97, 131.82, 126.40, 125.94, 125.57, 124.47, 63.74, 57.23, 53.02, 42.80, 42.60, 35.64, 27.81, 26.36, 26.19, 23.45, 21.21, 20.77, 19.53; ESI-HRMS m/z:calcd for C_27_H_32_N_2_O[M + 1]+ 401.2812, found 401.2866. 

#### 4.3.8. 14-[1-(4-Trifluoroethoxyl) Naphtholenyl] Matrine (ZS-10)

1,1-Dichloromethyl ether 7.47 g (65 mmol) was dissolved in 30 mL of dichloromethane under nitrogen protection at 0 °C. Anhydrous tin tetrachloride 16.94 g (65 mmol) was added dropwise with slow stirring and maintained at 0 °C for 1 h. When the solution turned light yellow, 1-fluoronaphthalene was dissolved in 20 mL dichloromethane and injected into the reaction solution, followed by slow heating of the reaction solution from ice bath conditions to room temperature with overnight stirring. The organic phase was separated, washed with water several times, and dried with anhydrous sodium sulphate under reduced pressure The crude product was concentrated to 8.86 g and purified by rapid silica gel column chromatography (eluent: n-hexane) to give 8.83 g of white solid in 93% yield. (4-fluoro-1-naphthaldehyde).

Subsequently, 20 mmol (0.64 g) of 4-fluoro-1-naphthaldehyde was dissolved in 20 mL of DMSO, and 58 mmol (1.4 g) of sodium hydride and 20 mmol of phenol were added. The reaction mixture was heated to 70 °C for 5 h, then filtered under reduced pressure and concentrated. The product was purified by silica gel column chromatography to yield 0.46 g as a white powder.

In the next step, ZS-10 was prepared by reference to the synthesis of ZS-1.

White powder, total yield: 44.0%, m.p.204.5–206.9 °C; purity by HPLC: 96.9%. ^1^H NMR (500 MHz, Chloroform-d) δ: 8.24(dd, J = 7.4, 1.6 Hz, 1H), 7.63(dd, J = 7.3, 1.7 Hz, 1H), 7.53(td, J = 7.5, 1.6 Hz, 1H), 7.48(td, J = 7.4, 1.6 Hz, 1H), 7.37(d, J = 1.3 Hz, 1H), 7.33(d, J = 7.5 Hz, 1H), 7.00(d, J = 7.5 Hz, 1H), 5.20~5.04(m, 2H), 3.98 (dd, J = 12.4, 6.6 Hz, 1H), 3.07~2.90(m, 4H), 2.19(dtd, J = 14.3, 7.1, 1.1 Hz, 1H), 2.15~2.03(m, 3H), 1.87~1.78(m, 1H), 1.72~1.61(m, 4H), 1.56~1.44 (m, 5H), 1.44~1.32(m, 1H), 1.18~1.03(m, 2H); ^13^C NMR (125 MHz, Common NMR Chloroform-d) δ 172.97, 155.81, 133.98, 133.50, 132.48, 130.08, 129.09, 128.54, 126.80, 126.22, 125.40, 123.55, 109.15, 57.11, 57.04, 56.27, 47.17, 42.39, 35.84, 27.71, 25.84, 23.90, 23.58, 21.78, 21.37.

#### 4.3.9. 14-[1-(4-(4’-F)-Naphthylmethoxy)naphthalenyl]matrine (ZS-11)

Light yellow powder, total yield: 56.0%, m.p.231.3~234.0 °C; purity by HPLC: 97.3%. ^1^H NMR (600 MHz, Chloroform-d) δ: 8.28~8.25(m, 1H), 8.24~8.21(m, 1H), 8.19(s, 1H), 8.14(dt, J = 8.9, 1.6 Hz, 1H), 8.00(dt, J = 8.4, 1.0 Hz, 1H), 7.62(dddd, J = 15.0, 8.3, 6.2, 4.8 Hz, 3H), 7.54(ddd, J = 8.3, 6.8, 1.4 Hz, 1H), 7.45(ddd, J = 8.2, 6.8, 1.2 Hz, 1H), 7.30(dd, J = 7.9, 1.0 Hz, 1H), 7.19(dd, J = 10.2, 7.7 Hz, 1H), 7.07~7.03(m, 1H), 5.65(s, 2H), 4.59(dd, J = 12.7, 4.4 Hz, 1H), 4.00(ddd, J = 10.6, 8.3, 5.3 Hz, 1H), 3.28(t, J = 12.6 Hz, 1H), 2.86(dd, J = 24.8, 11.4 Hz, 2H), 2.81~2.73(m, 1H), 2.43(dddd, J = 14.9, 11.1, 3.9, 2.0 Hz, 1H), 2.20~2.16(m, 1H), 2.15~2.07(m, 1H), 2.06~1.96(m, 3H), 1.89(d, J = 12.7 Hz, 2H), 1.84~1.76(m, 2H), 1.70~1.56(m, 3H), 1.54~1.39(m, 3H); 19F NMR (565 MHz, Chloroform-d) δ: -121.96; ^13^C NMR (151 MHz, Chloroform-d) δ: 164.91, 160.08, 158.40, 154.51, 133.15, 133.06, 132.49, 131.91, 128.24, 127.49, 126.97, 126.83, 126.30, 125.69, 125.47, 124.81, 124.17, 124.06, 123.93, 122.49, 121.25, 108.81, 108.68, 104.40, 68.59, 63.84, 58.47, 57.30, 53.05, 42.84, 42.67, 35.68, 27.84, 26.44, 26.29, 23.55, 21.25, 20.84, 18.44; ESI-HRMS m/z:calcd for C_28_H_34_N_2_O_3_[M + 1]+ 561.3113, found 561.3140.

#### 4.3.10. 14-(1-Naphthoxynaphthalenyl) Matrine (ZS-12)

Next, 1.13. g (6.5 mmol) of 4-fluoro-1-naphthaldehyde and 0.816 g (7.2 mmol) of α-naphthol were dissolved in 20 mL of acetonitrile. Then, 1.56 mL (7.8 mmol) of 2-tert-butyl-1,1,3,3-tetramethylguanidine was added at room temperature, then heated to 70 °C for 1 h. After cooling to room temperature, 65 mL of 1 M hydrochloric acid was added. The organic phase was combined, washed with water, dried over anhydrous sodium sulphate, concentrated under reduced pressure, and purified using silica gel column chromatography (eluent: V hexane: V ethyl acetate = 4:1) resulting in 1.84 g of a yellow solid product with 95% yield. (4-(1-Naphthyloxy)-1- naphthaldehyde)

Next, **ZS-12** was prepared by referring to the synthesis of ZS-1.

White powder, total yield: 67.0%, m.p.228.6~230.2 °C; purity by HPLC: 96.3%. ^1^H NMR (600 MHz, Chloroform-d) δ: 8.42(dd, J = 8.2, 1.4 Hz, 1H), 8.28(d, J = 8.3 Hz, 1H), 8.22(d, J = 9.3 Hz, 1H), 8.11~8.05(m, 1H), 7.93(d, J = 8.3 Hz, 1H), 7.71~7.66(m, 1H), 7.62~7.49(m, 4H), 7.40(dd, J = 9.8, 6.0 Hz, 1H), 7.23~7.16(m, 1H), 7.00(dd, J = 7.6, 0.9 Hz, 1H), 6.84(d, J = 7.8 Hz, 1H), 4.60(dd, J = 12.8, 4.4 Hz, 1H), 4.10~3.96(m, 1H), 3.30(t, J = 12.7 Hz, 1H), 3.13~3.02(m, 0H),2.90(dd, J = 23.4, 11.4 Hz, 2H), 2.79~2.67(m, 1H), 2.39(dddd, J = 14.8, 11.1, 3.7, 1.8 Hz, 1H), 2.26~2.15(m, 1H), 2.05(d, J = 25.0 Hz, 5H), 1.92~1.75(m, 3H), 1.75~1.54(m, 2H), 1.54~1.38(m, 3H); ^13^C NMR (151 MHz, Chloroform-d) δ: 164.76, 153.76, 152.98, 135.04, 133.39, 132.43, 132.27, 128.64, 127.90, 127.00, 126.90, 126.69, 126.33, 126.15, 125.88, 125.18, 123.70, 122.42, 122.00, 113.84, 111.58, 63.83, 60.41, 57.19, 52.99, 42.78, 42.63, 35.57, 29.70, 27.73, 26.27, 23.47, 21.09, 20.69; ESI-HRMS m/z:calcd for C_27_H_32_N_2_O_2_[M + Na]+ 583.3525, found 583.3555.

#### 4.3.11. 14-[1-(2,3-Dimethoxy) Naphthalenyl] Matrine (ZS-13)

White powder, total yield: 42.0%, m.p.174.0~174.3 °C; purity by HPLC: 95.9%. ^1^H NMR (600 MHz, Chloroform-d) δ: 7.88(s, 1H), 7.74(d, J = 8.3 Hz, 1H), 7.69(d, J = 8.1 Hz, 1H), 7.40~7.35(m, 1H), 7.31(ddd, J = 8.3, 6.8, 1.4 Hz, 1H), 7.14(s, 1H), 4.55(dd, J = 12.6, 4.4 Hz, 1H), 3.97(s, 5H), 3.76(s, 3H), 3.25(t, J = 12.6 Hz, 1H), 2.88~2.75(m, 3H), 2.34(ddd, J = 15.1, 7.0, 3.6 Hz, 1H), 2.13(d, J = 15.9 Hz, 2H), 2.07~1.88(m, 2H), 1.88~1.69 (m, 4H), 1.66~1.50(m, 2H), 1.50~1.28(m, 4H); ^13^C NMR (151 MHz, Chloroform-d) δ: 164.28, 151.95, 146.59, 134.89, 131.08, 127.89, 127.58, 126.78, 125.95, 125.44, 125.05, 124.08, 106.94, 63.75, 61.08, 57.22, 55.63, 53.22, 42.81, 42.52, 35.59, 27.79, 26.33, 25.70, 24.03, 21.18, 20.7; ESI-HRMS m/z:calcd for C_28_H_34_N_2_O_3_[M + 1]+ 447.2904, found 447.2922.

#### 4.3.12. 14-[2-(6-Benzyloxy) Naphtholenyl] Matrine (**ZS-17**)

White powder, total yield: 51.0%, m.p.175.3~178.1 °C; purity by HPLC: 98%. ^1^H NMR (600 MHz, Chloroform-d) δ: 7.87(s, 1H), 7.77(d, J = 8.9 Hz, 1H), 7.75~7.70(m, 2H), 7.53~7.49(m, 2H), 7.46~7.41(m, 3H), 7.39~7.34(m, 1H), 7.27~7.22(m, 2H), 5.21(s, 2H), 4.55(dd, J = 12.7, 4.5 Hz, 1H), 4.00(ddd, J = 10.9, 8.0, 5.1 Hz, 1H), 3.26(t, J = 12.7 Hz, 1H), 2.99(dddd, J = 14.9, 7.2, 3.8, 1.4 Hz, 1H), 2.86(dd, J = 28.6, 11.3 Hz, 2H), 2.61(dddd, J = 14.8, 10.7, 4.0, 2.0 Hz, 1H), 2.14(ddt, J = 17.5, 9.0, 3.6 Hz, 2H), 2.06~1.95(m, 2H), 1.94~1.88(m, 1H), 1.89~1.73(m, 3H), 1.72~1.63(m, 1H), 1.57(dddd, J = 17.8, 13.6, 7.9, 4.0 Hz, 3H), 1.46(dddd, J = 22.7, 18.2, 9.4, 5.6 Hz, 3H); ^13^C NMR (151 MHz, Chloroform-d) δ: 164.95, 157.27, 136.75, 134.60, 133.86, 131.78, 130.52, 129.77, 128.72, 128.64, 128.07, 128.00, 127.56, 126.64, 119.47, 107.08, 70.09, 63.80, 57.30, 52.88, 42.87, 42.62, 35.70, 27.82, 26.46, 25.87, 23.31, 21.24, 20.84; ESI-HRMS m/z:calcd for C_28_H_34_N_2_O_3_[M + 1]+ 464.2917, found 464.2864.

#### 4.3.13. 14-[1-(3-Methoxy) Naphtholenyl] Matrine (ZS-19)

White solid, total yield: 43.0%, m.p.175.5~177.3 °C; purity by HPLC: 96.4%. ^1^H NMR (600 MHz, Chloroform-d) δ: 8.09(s, 1H), 7.89(d, J = 9.0 Hz, 1H), 7.82~7.78(m, 2H), 7.45(ddd, J = 8.4, 6.7, 1.3 Hz, 1H), 7.36(ddd, J = 7.9, 6.7, 1.1 Hz, 1H), 7.12(d, J = 9.0 Hz, 1H), 4.59(dd, J = 12.6, 4.4 Hz, 1H), 4.00~3.93(m, 1H), 3.92(s, 3H), 3.26(t, J = 12.6 Hz, 1H), 2.89~2.78(m, 2H), 2.32~2.25(m, 1H), 2.18~2.13(m, 2H), 2.08~1.96(m, 4H), 1.89~1.82(m, 2H), 1.82~1.75(m, 2H), 1.69~1.54(m, 3H), 1.52~1.45(m, 2H), 1.45~1.36(m, 2H); ^13^C NMR (151 MHz, Chloroform-d) δ: 164.75, 154.37, 134.65, 132.83, 129.56, 128.78, 128.16, 128.12, 126.49, 124.81, 123.65, 119.19, 113.37, 63.84, 57.30, 56.16, 53.24, 44.83, 42.51, 35.61, 27.85, 26.43, 25.78, 24.09, 21.25, 20.81; ESI-HRMS m/z:calcd for C_27_H_32_N_2_O_2_[M + 1]+ 417.2849, found 417.2820.

### 4.4. MTT Assay

BEL-7402, MCF-7, SGC-7901, and H460 cells were seeded in 96-well plates at a density of 3000 cells/well. Twenty-four hours later, the cells were treated with **ZS17** at the indicated concentrations for 24, 48, and 72 h. MTT was then added to the 96-well plate and incubated at 37 °C for 4 h. Next, 150 mL DMSO was added to each well and gently shaken for 15 min. Finally, the optical density was measured using a microplate reader (PerkinElmer, Melville, NY, USA) at 490 nm. 

### 4.5. Determination of LDH Activity

Cells were seeded into 96-well plates at a density of 5000 cells/well and incubated in DMEM and RPMI 1640 for 24 h. **ZS17** (0, 2, 4, and 6 μM) was then added to the cells. LDH activity was determined in the supernatant using an LDH kit, following the manufacturer’s instructions on the kit manual.

### 4.6. Wound-Healing Assay

BEL-7402 and HepG2 cells were added to 6-well plates at 5 × 10^4^ cells per well. When the cells reached 70% confluence, a straight line was scratched across the cells using a 100 μL pipette. Cells were washed off and incubated in culture medium containing **ZS17** (0, 2, 4, and 6 μM) for 48 h. Cells in the same field of view were photographed at 24 h intervals, and the mean migration rate was calculated.

### 4.7. Transwell Migration Assay

Cells (2 × 10^4^) in serum-free culture medium were inoculated into the upper chamber. Serum-containing complete culture medium was added to the bottom chamber, and **ZS17** (0, 2, 4, and 6 μM) was added to inhibit cell migration. Twenty-four hours later, cells on the surface were wiped away, and lower surface cells were fixed in methanol, stained with crystal violet, and counted.

### 4.8. Cell Colony Formation Assay

Approximately 600 cells were added to each well of a 6-well culture plate. Five to six days later, cells were fixed in 4% paraformaldehyde for 25 min and stained with crystal violet for 25 min. After three washes, images were acquired using a colony-counting analysis system.

### 4.9. Measurement of Intracellular ROS and GSH

Cells were incubated with **ZS17** (0, 2, 4, and 6 μM) for 48 h and then the cells were washed with PBS, followed by the addition of 10 μM DCFH-DA solution, incubated for 30 min, and analysed by flow cytometry.

Furthermore, the cells were incubated with different concentrations of **ZS17** for 48 h, then lysed and subjected to GSH assays. Cell supernatants were collected, and GSH content was assayed using a commercial kit (Beyotime, Nanjing, China).

### 4.10. Determination of Effect of ZS-17 on Mitochondrial Membrane Potential

After 24 h of culture, BEL-7402 or HepG2 cells were seeded into 6-well plates at a density of 4 × 10^5^ cells/well. The cells were treated with graded concentrations of **ZS17** for 48 h and stained with JC-1 working solution for 30 min at 37 °C in the dark. The cells were then washed twice with staining buffer and analysed using confocal laser scanning microscopy.

### 4.11. Measurement of Oxygen Consumption Rate

A SeaHorse XF24 cellular outflow analyser (SeaHorse Bioscience, North Billerica, MA, USA) was used to detect real-time integrated cellular oxygen consumption rate (OCR). Then, 1.2 × 10^4^ cells were inoculated into hippocampal custom cell plates and **ZS17**-treated BEL-7402 and HepG2 cells for 48 h. After baseline measurements, OCR was detected with sequential injections of oligomycin (an ATP synthase inhibitor; 1 μM), FCCP (uncoupler; 0.5 μM), rotenone (complex I inhibitor;1 μM), and antimycin A (complex III inhibitor; 1 μM).

### 4.12. Flow Cytometry Analysis for Cell Cycle and Apoptosis

The cells were inoculated in six-well plates (2 × 10^5^ cells/well) and incubated with **ZS17** (0, 2, 4, and 6μM) for 48 h. The cells were washed with PBS and fixed with 75% ethyl alcohol at 4 °C overnight. The cells were subsequently stained with propidium iodide (BD, Franklin Lakes, NJ, USA) and injected into a flow cytometer for analysis. Cells were digested and washed three times, and PI and Annexin-V (BD, Franklin Lake, NJ, USA) were co-stained for 15 min and analysed for apoptosis. The apoptosis rate was determined using flow cytometry.

### 4.13. Western Blot Analysis

Equal amounts of protein were dissolved in SDS-PAGE buffer and transferred to 0.45 PVDF membranes. After 45 min of blocking with QuickBlock™ Closure Buffer (Beyotime, Nanjin, China), the membrane was incubated with the primary antibody overnight at 4 °C and then with the secondary antibody for 50 min at room temperature. Visualisation was performed using the Bio-RAD ECL detection system (three independent experiments).

### 4.14. Reverse Transcription-Quantitative Polymerase Chain Reaction

Total RNA from **ZS17**-treated BEL-7402 or HepG2 cells was extracted using the GoScrip TM Reverse Transcription System (Promega, Beijing, China) and treated for 48 h. RNA was then converted into cDNA using the GoScript TM Reverse Transcription System (Promega). The following primers were used: P53, P21, Bax, Bcl-2, caspase-3, 8, 9, cytochrome c, CDK2, PARP, JNK, and β-actin (all synthesised by Generay Co., Ltd.). The relative expression of each gene was normalised to that of β-actin (*ACTB*), used as an internal standard. Relative quantification of gene expression was performed. All analyses were performed in triplicate for each sample.

### 4.15. Hematoxylin and Eosin (H&E) Staining

To evaluate **ZS17**-induced damage to the major organs of nude mice, liver, heart, spleen, lung, and kidney tissues of mice were harvested, fixed with xylene, and embedded in paraffin. Sections were stained with H&E according to standard methods. The slides were observed under a microscope (Olympus, VS200, Tokyo, Japan), and representative images were acquired.

### 4.16. Immunofluorescence

Tumour specimens from nude mice were fixed overnight in 4% paraformaldehyde and embedded in paraffin. The samples were incubated with primary antibodies against cytochrome c (1:100, ABCAM) and P53(1:100, ABCAM) overnight at 4 °C. The samples were then incubated with a secondary anti-rabbit antibody (ABCAM) for 2 h at ambient temperature. Samples were visualised with an Olympus VS200 fluorescence microscope.

### 4.17. Establishment of Zebrafish Embryo Liver Tumour Model

HepG2 cells were resuspended in DMEM and labelled with CM-DIL for red fluorescence. Then, 50 nL of HepG2 cells was injected into the zebrafish yolk gap using a micro-injector to establish a zebrafish embryonic liver tumour model. Zebrafish, a 48 h-old model of liver tumour, were cultured in 24-well plates (one tail per well) with 600 μL of **ZS17** (0, 2, 4, and 6 μM) in water. HepG2 cell migration in zebrafish was observed using fluorescence microscopy and photographed. The specific animal use committee protocol for the zebrafish embryo experiment was GXLESCMM-20211205.

### 4.18. Animal Experiments

Five-week-old nude mice were purchased from Cave Laboratories (Changzhou, China). HepG2 cells were subcutaneously inoculated into mice (1 × 10^7^ cells). Mice with a tumour diameter of 100 mm^3^ were randomly divided into control and treatment groups (n = 6/group). Matrine (40 mg/kg), sorafenib (15 mg/kg), and **ZS17** (40 mg/kg) were administered via oral gavage for 24 days. The tumour sizes and body weights of nude mice were measured and recorded once every other day. At the end of the experiment, the mice were sacrificed and photographed. These experimental procedures were approved by the Animal Research Ethics Committee of La Muda, Jiangsu Province, China. The animal use committee protocol number for the animal experiments was IACUC-20220401.

### 4.19. Statistical Analysis

Statistical analyses were performed using GraphPad 8.0.2 software (GraphPad Inc., San Diego, CA, USA) and *p* < 0.05 was considered statistically significant. The results are presented as mean ± standard deviation (SD).

## 5. Conclusions

In summary, we designed and synthesised 12 novel matrine derivatives. Their anti-proliferative activities were evaluated against liver, breast, gastric, and lung cancers. Among them, **ZS17** exerted a more potent anti-tumour effect than matrine by activating the ROS-JNK-P53 signalling pathway. The activated ROS-JNK-P53 pathway further induces mitochondrial dysfunction and apoptosis in cancer cells. Although we identified that the ROS-JNK-P53 signalling pathway plays a partial role in the induction of apoptosis in BEL-7402 and HepG2 cells by **ZS17**, we have not confirmed whether the ROS-JNK-P53 signalling pathway is associated with other physiological processes. A potential mechanism by which **ZS17** affects ROS production and the mitochondrial pathway is shown in Figure 9. Our results suggested that **ZS17** could be a potentially effective approach for treating liver cancer.

## Figures and Tables

**Table 1:** The IC50 (μM) value of compounds against human tumor cell lines for **48** h.

Compounds	IC50 (µM)			
	BEL-7402	MCF-7	SGC-7901	H460
ZS-01	11.5 ± 2.4	11.1 ± 4.2	13.5 ± 5.4	11.4 ± 3.7
ZS-04	13.5 ± 1.5	14.8 ± 1.2	12.7 ± 1.3	15.5 ± 1.4
ZS-05	4.2 ± 0.3	9.3 ± 1.4	7.4 ± 0.5	9.1 ± 1.5
ZS-07	1.8 ± 0.2	2.4 ± 0.3	1.6 ± 0.3	2.6 ± 0.5
ZS-08	9.2 ± 1.4	10.2 ± 1.4	9.4 ± 1.2	11.8 ± 1.5
ZS-09	5.1 ± 1.4	8.9 ± 3.1	5.9 ± 2.1	9.5 ± 2.3
ZS-10	1.5 ± 0.3	2.2 ± 0.8	1.9 ± 0.5	2.1 ± 1.2
ZS-11	6.6 ± 0.6	9.3 ± 1.2	7.4 ± 0.6	8.1 ± 0.9
ZS-12	5.8 ± 0.7	4.3 ± 0.9	6.2 ± 0.7	7.4 ± 0.6
ZS-13	6.7 ± 0.9	12.8 ± 1.4	7.4 ± 0.8	10.4 ± 1.7
ZS-17	3.0 ± 0.4	3.2 ± 0.3	3.15 ± 0.4	3.5 ± 2.1
ZS-19	8.2 ± 1.4	9.3 ± 2.8	9.8 ± 3.9	9.2 ± 1.7
Matrine	2057 ± 192	2497 ± 208	1380 ± 116	2849 ± 268
Sorafenib	0.568 ± 0.056	1.02 ± 0.16	0.756 ± 0.042	1.122 ± 0.201

## Data Availability

Data available in a publicly accessible repository. The data presented in this study are openly available in https://www.jianguoyun.com/p/DSHmXtEQuY_OCRj__-wEIAA (accessed on 20 November 2022).
